# Behind the Scenes of Developmental Language Disorder: Time to Call Neuropsychology Back on Stage

**DOI:** 10.3389/fnhum.2018.00517

**Published:** 2019-01-09

**Authors:** Ekaterina Tomas, Constance Vissers

**Affiliations:** ^1^National Research University Higher School of Economics, Moscow, Russia; ^2^Behavioural Science Institute, Radboud University Nijmegen, Nijmegen, Netherlands; ^3^Royal Dutch Kentalis, Sint-Michielsgestel, Netherlands

**Keywords:** Developmental Language Disorder, Specific Language Impairment, neuropsychology, executive functions, attention, perception

## Abstract

Although the Developmental Language Disorder (DLD), also known as Specific Language Impairment in children has been the focus of unceasing scientific attention for decades, the nature and mechanisms of this disorder remain unclear. Most importantly, we still cannot reliably identify children requiring urgent intervention among other ‘late talkers’ at an early age and understand the high prevalence of comorbidity with psychiatric phenomena such as Autism Spectrum Disorder. One of the main reasons for this is the traditional ‘diagnosis-by-exclusion,’ resulting in heterogeneity of the DLD population. This paper proposes an alternative approach to the diagnosis, treatment and research of DLD, claiming that it is these children’s multiple deficits in neuropsychological development, which impede the spontaneous acquisition of their first language. Specifically, this review of the state-of-the-art in DLD research demonstrates deep and systematic interconnections between the speech and other higher cognitive functions developing in early childhood, including perception, attention and executive functions. In the proposed framework, speech is, therefore, considered as one of neuropsychological abilities, and the delay in its development is explained by other neuropsychological deficits, resulting in highly individual clinical profiles. By considering DLD as a complex neuropsychological syndrome, whose successful treatment depends on a holistic approach to diagnosis and intervention, we may significantly increase the efficacy of speech therapy, and also better understand the flexibility of the developing brain, its compensatory mechanisms and hence the comorbidity of DLD with psychiatric symptoms. Implications for using this paradigm in future scientific research are discussed.

## Introduction

Developmental Language Disorder (DLD) is characterized by the absence of speech in children despite their normal non-verbal IQ, no primary physical disabilities, neurological disorder or mental illness (Leonard, 2008; Reilly et al., 2014; Bishop et al., 2016, 2017). It is observed in approximately 5–10% of the population (Tomblin et al., 1997a; Law et al., 2000), and possibly because of the high proportion of children suffering from DLD, this disorder has long been the focus of attention in scientific research (e.g., Laurence and Karla, 1981; Rice and Wexler, 1996; Cleave and Rice, 1997; Leonard et al., 1997; Conti-Ramsden et al., 2001; Rice and Wexler, 2001; Rice et al., 2004; Marinis, 2011; Henry et al., 2012; Archibald et al., 2013; Kapalková, 2013; Vissers et al., 2015; Vissers and Koolen, 2016; Tomas et al., 2017) and clinical studies (e.g., Ukoumunne et al., 1999; Yoder and McDuffie, 2002; Law et al., 2003; Warren et al., 2007; Strong et al., 2011; Zeng et al., 2012; Smith-Lock et al., 2013; Law et al., 2017). It has been shown that DLD can be reliably diagnosed after the age of 4 years (Whitehurst and Fischel, 1994; Paul, 1996; Rescorla et al., 2000) and that it can be roughly characterized as a lag of about 2 years in the development of language abilities (Rice et al., 2006). A recent Delphi Consensus Study has additionally pointed out some specific indicators of atypical language development in 4–5-year-old children, including inconsistent or abnormal verbal interaction and at most three word utterances (Bishop et al., 2016, 2017). Importantly, children diagnosed with DLD as preschoolers later on often have difficulties in their social-emotional development (St Clair et al., 2011; Vissers and Koolen, 2016; Forrest et al., 2018) and they also demonstrate lower levels of school performance. The latter, at least in part, can be attributed to the fact that a large proportion of children with DLD also develop dyslexia (Bishop and Snowling, 2004; Rakhlin et al., 2013). It has further been shown that poor expressive abilities in early childhood are the best predictor of reading problems and dyslexia in school-aged children (Lyytinen et al., 2015; also see discussion in Eklund et al., 2018). It appears, therefore, that these reciprocal connections between children’s limited expressive abilities during preschool years, and their reading difficulties and poorer academic performance at school, place children with DLD at a further disadvantage compared to their peers.

Over the past several decades both genetic (Bishop et al., 1995, 2006; Tomblin et al., 1997b; Dale et al., 1998; Tomblin and Buckwalter, 1998; Bishop, 2009; Graham and Fisher, 2013; Rice, 2013) and environmental risk factors for DLD (Tomblin et al., 1997b; Bishop, 2009; Law et al., 2012) have been identified. Despite years of research, however, the underlying *causes* for this disorder are still not understood. The most serious problem in this respect poses the great amount of *heterogeneity* observed in this population, which suggests that DLD is probably not a single type of disorder, but an umbrella term for a variety of deficits in the domain of language acquisition (Bishop, 1994; Conti-Ramsden and Botting, 1999). Purely linguistic accounts of DLD claim that this disorder is specific to language, suggesting that other neuropsychological processes remaining largely intact (Rice and Wexler, 1996; van der Lely, 2005; Stavrakaki, 2006; Rothweiler et al., 2012). These linguistic approaches focus on establishing the various clinical markers of DLD in the language domain, which could be targeted during speech screening and intervention.

Alternative accounts of DLD have observed that children suffering from this impairment often have additional neuropsychological deficits accompanying their language problems. However, there has traditionally been a strong tendency to search for a *single* deficient neuropsychological mechanism underlying DLD, and thus the main body of research has compared children with and without DLD on the basis of either their working memory (WM) capacity (Gathercole and Baddeley, 1990; Bishop et al., 1996; Archibald and Gathercole, 2006; Falcaro et al., 2008), or auditory perception abilities (Tallal and Piercy, 1973; Bishop et al., 1999c; Wright et al., 2000; Ziegler et al., 2005), or sustained attention (Spaulding, 2008; Finneran et al., 2009; Ebert and Kohnert, 2011), etc. In contrast, it has recently been put forward that DLD is not only closely associated with neuropsychological deficits, but occurs when at least two cognitive processes are disrupted (Bishop, 2006). This observation is in line with what has long been claimed by the proponents of neuropsychological approach to speech pathology going back to the 1930’s (Vygotsky, 1934) and later expanded in the 1950–1960’s (Luria, 1962, 1966). Neuropsychology is concerned with the ‘behavioral expression of brain dysfunction’ (Lezak et al., 2004) and it thus suggests deep interconnections between the various higher cognitive processes, including, for example, language and executive functions (EFs). Within this framework, the causes underlying the observed behavioral problems are thus thought to be rooted in multiple neurophysiological deficits.

In the speech and language domain, therefore, the ability to spontaneously acquire a language relies on the child’s neuropsychological skills, and thus the absence of speech needs to be considered as a *symptom* of their underdeveloped neuropsychological functions rather than an isolated deficit. This is supported by empirical evidence showing that language learning deficits are often observed across different clinical populations, including children with hearing loss (Briscoe et al., 2001; Moeller et al., 2010), children with ADHD (Geurts and Embrechts, 2008; Green et al., 2014), Autism Spectrum Disorder (Koolen et al., 2012) as well as those with mental retardation (Marrus and Hall, 2017) or cerebral palsy (Hustad et al., 2014). In these groups of children the absence of speech is clearly secondary to another pathology, such as impaired auditory perception in children with hearing loss, or impaired executive control and social-emotional deficits in the ADHD and Autism Spectrum Disorder. What is not so clear is whether language deficits in DLD are *also* secondary – perhaps, not to a single primary disorder, but to a combination of underdeveloped higher cognitive functions. If this is the case, then our aim should be to identify at least some typical *combinations* of neuropsychological deficits in children with DLD and focus on the associations between these children’s neuropsychological profiles and their corresponding patterns of language-learning difficulties. Despite the existing empirical evidence, there is currently no reliable method correlating the child’s neuropsychological and language profiles. However, it seems that targeting those primary deficits in cognitive processes, which impede the child’s spontaneous acquisition of their first language, would be the first step toward increasing the efficacy of assessment and intervention. The following section of the paper, therefore, gives an up-to-date overview of what is known about cognitive performance by children with DLD.

## Perception

Perception is a multi-dimensional ability, which can be explored not only across different domains (e.g., auditory, visual, tactile, etc.), but also in terms of the types of information processed within each domain. For the purposes of this paper, we will focus on the various aspects of auditory perception, since the perception in the visual domain appears to play a secondary role in the acquisition of oral speech (Levina, 1951; Guenther and Hickok, 2015; Guenther, 2016). It has long been observed that at least some of the children with severe language-learning problems demonstrate deficits in their auditory perception skills. Specifically, back in early 1950’s it has been shown that despite these children’s normal hearing abilities, they fail to perceive linguistically meaningful contrasts (Levina, 1951). In 1970’s, it has first been proposed that the perceptual deficit might be more generic in its nature and that children with DLD have difficulties in perceiving the various acoustic properties of non-verbal auditory signals as well (Tallal and Piercy, 1973; Tallal et al., 1985). It has also been found that the skills in perceiving non-verbal auditory information can be trained, thus improving overall language abilities (Tallal et al., 1996).

Both verbal and non-verbal auditory information can be described in relation to four physical parameters: duration, frequency, amplitude and phase. The first three are most relevant for studying atypical speech development because in the language domain they represent acoustic features used for discrimination of phonemes and words. Duration, or temporal processing, has been thoroughly examined in classical studies by Tallal and her colleagues, demonstrating that children with DLD have difficulties discriminating and reproducing tones of short duration, as well as determining the order of rapidly changing elements in a sequence (Tallal and Piercy, 1973; Tallal, 1980; Tallal et al., 1985). A more recent study, reporting on a larger group consisting of 16 children with DLD, has expanded these observations by identifying two subgroups of children with DLD: those with poor and with normal temporal resolution abilities (Ahmmed et al., 2006). In addition, the study discusses the interaction between these children’s temporal resolution and frequency perception abilities, suggesting a compensatory mechanism in children with DLD. Specifically, the subgroup of children who demonstrated poorer temporal resolution abilities showed greater frequency sensitivity.

The perception of frequency has also been studied independently of temporal effects, showing that children with DLD are less sensitive in perceiving voicing contrasts (e.g., /p/–/b/, as in a minimal pair ***p**at – **b**at*) compared to their typically developing (TD) peers (Ziegler et al., 2005, 2011). These deficits in perceiving the elements of short duration and different frequency suggest that these children with DLD would have problems efficiently processing rapid speech, extracting phonological elements of short duration (e.g., grammatical morphemes in English, as in *He run**s***) and also in forming stable phonological representations of words, particularly if they form minimal pairs, as in ***s**eal – **z**eal*, or have similar sounding counterparts, as in *agile – **fr**agile*.

The research on the perception of amplitude focuses on the sensitivity to suprasegmental speech rhythm and stress patterns in children with DLD. Very few studies have explored these problems, and only in children’s perception of verbal signals. However, it has been shown that children with DLD tend to have decreased sensitivity to amplitude envelope rise time in interaction with both frequency (Richards and Goswami, 2015) and duration (Corriveau et al., 2007) of the signal. In the language domain, this means that children with DLD are likely to be less sensitive to lexical and phrasal stress and its violations.

However, the original hypothesis that speech deficits in children with DLD arise from their acoustic processing limitations has been challenged in more recent twin studies. Specifically, the authors have found no significant relationship between non-verbal and verbal auditory processing abilities in children with DLD (Bishop et al., 1999a,b,c; Bailey and Snowling, 2002). These findings have started a long-standing debate with some studies showing that at least some children with DLD have deficits in non-linguistic auditory perception and that their difficulties are not specific to language (Wright et al., 1997, 2000; Hill et al., 2005; Ziegler et al., 2005, 2011; Corriveau et al., 2007; Vandewalle et al., 2012; Richards and Goswami, 2015). These disparate results are probably due to heterogeneity of the DLD population, particularly since the majority of these studies report on a small number of participants. It seems reasonable to assume that a proportion of children with DLD might have limitations in the auditory perception domain as the primary source of their language difficulties. It thus appears that neuropsychological assessment for children with DLD should include screening for possible deficits in auditory perception of both verbal and non-verbal signals.

## Attention

**FIGURE 1:**
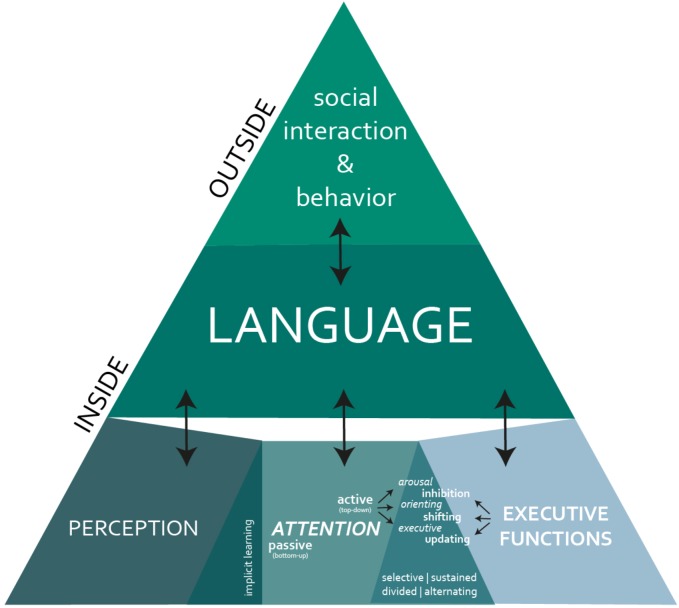
Neuropsychological perspective on cognitive and social functioning: the interplay between perception, attention and executive functions forms the foundation for language abilities. During social interaction, possible neuropsychological deficits are then behaviorally manifested through language as a proxy.

Attention is essential for (language) learning because it serves as a ‘filtering system’ for the constant stream of input information, thus allowing to process only its relevant features. Attention is closely associated with both perception and EFs (Johnston and Dark, 1986; Styles, 2006; also, see Figure 1). Specifically, like perception, attention can be an unconscious *passive* bottom-up process governed by our sensitivity to regularities in the input guided by our expectations and experience. For example, our attention is attracted by an unusual phenomenon, such as seeing a yellow leaf among green leaves on the pavement. This ability to unintentionally perceive regularities and deviations from patterns is closely associated with implicit learning (or so-called Statistical Learning) skills, which, due to their automatic nature, are believed to be one of the key leaning mechanisms across cognitive domains (Turk-Browne et al., 2005; Endress and Mehler, 2009; Romberg and Saffran, 2010; Siegelman et al., 2016), functioning from infancy (Saffran et al., 1996; Romberg and Saffran, 2010).

However, our attention can also be *actively* (i.e., consciously/intentionally) focused on some phenomenon, thus becoming a top–down process also known as attention control. For example, when we search for a birch tree leaf among other leaves on the pavement. Because attention control allows one to focus on a task and is involved in program selection, it is commonly listed among EFs (McCabe et al., 2010; Najdowski et al., 2014; Drigas and Karyotaki, 2017; also, see a more detailed discussion of this topic in the following section of the paper).

Over the years, several models have been proposed to describe how these types of processes are carried out in the brain. One of the best empirically supported frameworks is the recent component theory of attention (Mirsky et al., 1991; Gomes et al., 2000). This theory distinguishes several cortical sites orchestrating three types of behavior, associated with attention: alerting/arousal, orienting and executive attention (Posner and Petersen, 1990; Mirsky et al., 1991; Petersen and Posner, 2012). Alerting refers to the general physiological readiness to perceive and process stimuli (Gomes et al., 2000; Rueda et al., 2004; Petersen and Posner, 2012). It is associated with maintaining optimal vigilance and performance during tasks, and it is thus often recognized as an essential component of sustained attention (Ballard, 1996; Styles, 2006; Finneran et al., 2009; Posner, 2012; Langner and Eickhoff, 2013; Fortenbaugh et al., 2017). Orienting involves shifting attention to endogenous or exogenous cues (Corbetta and Shulman, 2002; Posner et al., 2006), which seems to be fundamental for unconscious/implicit learning mechanisms. Finally, executive attention refers to the ability to detect errors, resolve conflict among responses or inhibit responses (Posner et al., 2006; Petersen and Posner, 2012). This form of behavior, which has been originally proposed as part of the attention network (Posner and Petersen, 1990), is also often considered among EFs (see more on this in the discussion of inhibitory control in the “Executive Functions” section). In addition, in many models of attention executive attention is associated with selective attention, i.e., the ability to detect only relevant stimuli in the stream of input (Moray, 1959; Johnston and Dark, 1986; Wood and Cowan, 1995; Stevens et al., 2006; Shinn-Cunningham, 2008; Getzmann and Näätänen, 2015).

Several studies have attempted to explore these problems from developmental perspective (Ruff and Rothbart, 1996; Gomes et al., 2000; Rueda et al., 2004; Thillay et al., 2015; Suades-González et al., 2017), confirming that some types of attention like arousal and orienting systems form in early infancy, and finding mixed results for selective and sustained attention abilities (Gale and Lynn, 1972; Swanson, 1983; Gomes et al., 2000). The former is probably indicative of the innateness of some automatic/unconscious (bottom–up) attention mechanisms, such as those involved in Statistical Learning. More cognitively demanding and complex types of behavior, including controlled selective and sustained attention or divided attention, which requires simultaneous processing of multiple streams of information (Sohlberg and Mateer, 1989), are likely to develop gradually, with the maturation of the respective cortical areas. However, more research is needed to determine the developmental trajectories for these types of attention across auditory, visual and other domains (see for review: Gomes et al., 2000).

Regarding children with DLD, it seems that at least some of the bottom–up processes are impaired in this population. Specifically, the unconscious attention mechanisms associated with implicit learning tend to be weaker in these children compared to their TD peers (see for reviews: Lammertink et al., 2017; Zwart et al., 2017, 2018). However, it is not clear whether these poorer abilities in perceiving statistical regularities are domain-specific (e.g., visual vs. auditory statistical learning skills); and also if there is a difference between verbal and non-verbal types of tasks within the auditory domain. More cognitively demanding types of attention have been studied less systematically in children with DLD, often producing disparate results. Recent meta-analysis on sustained attention abilities in the DLD population, however, supports the idea that these children tend to have deficits in sustained attention across auditory and visual modalities, and that larger effect sizes can be found for auditory (both verbal and non-verbal) stimuli (Ebert and Kohnert, 2011). Similarly, in the selective attention domain, children with DLD demonstrate poorer performance during verbal and non-verbal auditory tasks (Stevens et al., 2006), but not in visual tasks (Spaulding, 2008).

To summarize, attention deficits could be one of the underlying causes of DLD. However, it is not yet clear how the various types of attention interact to meet cognitive demands for these children. For example, it is not known whether children with DLD develop some compensatory executive attention mechanisms if they have deficits in their implicit/unconscious attention abilities. In addition, more evidence is needed to understand the extent of their attention deficits across modalities, and also during more cognitively demanding types of tasks, involving alternating and divided attention.

## Executive Functions

Executive functioning can be defined as a top–down control of cognitive processes for goal achievement. Executive control is necessary in the regulation of more automatic processes (thoughts, behavior, emotion) in the service of a goal or to adjust to changing circumstances (Miyake et al., 2000). EFs are especially important in situations in which relying upon automatisms or impulses is unwise or even impossible, as in non-routine situations (Diamond, 2013). There is controversy over the specific components of executive functioning and the way they relate to each other (Barkley, 1997, 2012; Miyake et al., 2000). A well-established conceptualization of EFs is Miyake’s model, which considers it as a unitary construct with three separable major components: inhibition of prepotent responses (inhibiting), shifting between tasks or mental sets (shifting) and information updating and monitoring of WM representations (updating).

Inhibition refers to the ability to deliberately suppress dominant or automatic responses and to resist interference of distractors (Friedman and Miyake, 2004). Inhibitory control is important for learning as it helps maintain sustained and focused attention necessary for the acquisition of new skills and knowledge (see also section “Attention”). Inhibiting is also important for social functioning; for example, resisting impulses and temptations (e.g., waiting for your turn) is essential for establishing and maintaining social relationships (Tangney et al., 2004).

Shifting between tasks or mental sets involves the disengagement of a task set and the active engagement of a new task set. Shifting also involves the ability to switch between operations or mental sets. The ability to shift is strongly related to cognitive flexibility. It is essential to social-emotional functioning as people bring their own goals, impulses, desires, and emotions into social situations, which makes every social situation unique and often very complicated and unpredictable (Parsons and Mitchell, 2002).

Updating is the ability to actively manipulate the contents of WM and to monitor the incoming information with the aim of keeping track of which information is relevant and update items in WM with new, more relevant information. In conceptualizing WM, Baddeley’s multicomponent WM model is widely used. It comprises three subsystems governed by the central executive: the phonological loop, the visuo-spatial sketchpad, and the episodic buffer (Baddeley, 2000). The former two are ‘slave’-systems responsible for temporary storage of verbal and visuo-spatial information. The episodic buffer is proposed to integrate representations from WM, long-term memory and language processing systems. WM is essential for learning and also for social functioning because it subserves temporal processing of social information during interactions, keeping social goals actively in mind, retrieving social information from long-term memory, and selecting an appropriate social response (see also Vissers and Hermans, 2018).

Executive functions have been discussed extensively in investigating DLD over the past decade. Deficits or delays in the development of EFs in children with DLD have been reported for many components of the executive system, but in some more than in others (e.g., Hill, 2004; Bishop and Norbury, 2005; Castellanos et al., 2006). Specifically, studies on inhibitory control processes have reported consistent results, showing that children with DLD tend to be impaired in their inhibiting abilities (e.g., Bishop and Norbury, 2005; Marton et al., 2007; Pauls and Archibald, 2016). In particular, children with DLD are more susceptible to distraction (Lum and Bavin, 2007). Interestingly, this impaired performance of children with DLD has been described for the auditory distraction task regardless of whether a distractor was related or unrelated to the target stimuli. This suggests that children with DLD might have a generic distractor processing problem (Victorino and Schwartz, 2015).

With respect to cognitive flexibility, findings are mixed. Thus, some studies on shifting have not found any deficits in children with DLD (e.g., Kiernan et al., 1997; Im-Bolter et al., 2006), others have brought to light attentional shifting problems in addition to decreased cognitive flexibility (Marton, 2008). Overall, there is no consistent evidence indicating an impairment in shifting ability of the DLD population (e.g., Kapa and Plante, 2015), and more research is needed to explore the developmental trajectory of this ability and its role in language learning in both TD and DLD populations.

Studies on updating and WM in children with DLD have found limitations on both phonological and non-verbal WM tasks (Marton and Schwartz, 2003; and see also Archibald and Gathercole, 2006; Bishop, 2006; Montgomery et al., 2010; Duinmeijer et al., 2012). In line with these findings, Im-Bolter et al. (2006) have found impairments in children with DLD in updating the general WM content. In contrast, some studies examining non-verbal updating ability in DLD show conflicting results. Specifically, several studies report similar visuo-spatial updating performance of children with DLD and their TD peers (e.g., Lum et al., 2012). However, recent meta-analysis on visuo-spatial WM in DLD (Vugs et al., 2013) suggests that for this population WM deficits indeed extend to the non-verbal domain. Similarly, Henry et al. (2012) have observed differences in verbal and non-verbal updating ability between DLD and TD children after controlling for their non-verbal IQ, and also differences in non-verbal updating ability after controlling for their verbal IQ. This suggests that poorer non-verbal updating performance reflects a domain general updating deficit.

Importantly, it is often challenging to explore EFs in children under 4 years due to cognitive demands of the tasks. Thus, a lot of research in this area has so far focused on school-aged children, and more evidence is required to understand the role of EFs on language acquisition in children. Recent studies suggest that similarly to school-aged children with DLD, preschoolers with DLD tend to show difficulties in WM, inhibition and shifting, as revealed by both performance-based measures and behavioral ratings (Vissers et al., 2015). However, it is not yet clear to what extent this relationship between the developing EFs and language abilities in children is reciprocal, and to what extent it might be causal. It thus seems particularly important to explore the interconnections between children’s individual language profiles and EFs during typical development and as a part of the diagnosis for DLD.

## Discussion

This paper aimed at bringing together findings from different areas of neuropsychological research, exploring DLD and its underlying causes. It focused on how the various higher cognitive processes, including perception, attention, inhibition control, mental flexibility and WM may affect the spontaneous emergence of speech. Despite the observed inconsistencies across individual studies, overall there seems to be a strong association between DLD and the deficits in higher cognitive processes essential for normal language acquisition and functioning. In particular, there appears to be a continuous interplay between perception, attention, EF and language across childhood (see Figure 1 for a schematic representation of this neuropsychological perspective on cognitive and social functioning). This cognitive interplay might underlie problems in communication and social-emotional functioning observed in many children with DLD. From here, we propose that DLD needs to be treated as a complex neuropsychological syndrome during diagnosis and therapy. Specifically, it appears that diagnosis will benefit from screening for possible neuropsychological deficits underlying DLD, and that targeting these impaired neuropsychological abilities is likely to complement and enhance speech intervention.

Several important theoretical questions remain open, however. First, it is not clear if there is a straightforward correlation and interaction between different modalities (e.g., auditory vs. visual attention deficits). Second, it is not known whether for auditory information these deficits are specific to linguistic input or are more generic in their nature (e.g., verbal vs. non-verbal WM limitations). Third, little is known about how the various types of neuropsychological deficits interact, and how this may reflect on the child’s individual linguistic profile. Also, more information is needed to better understand the developmental trajectories of the higher cognitive processes observed in TD children. Thus, for example, it is not known whether a child with normal language skills may have deficits in one or more higher cognitive abilities; and if so, what compensatory mechanisms they develop to prevent these neuropsychological impairments from leading to DLD. Finally, it seems essential to resolve the problem of heterogeneity in the language abilities observed in the DLD population, which is likely to be due to variability in types of neuropsychological deficits impeding the spontaneous emergence of speech in these children (see Box 1). Thus, matching their individual language and neuropsychological profiles during diagnosis would allow selecting a more effective intervention program that would target specific deficient cognitive ability [e.g., WM training, attentional training, perceptual (rhythmic/music) intervention etc.]. This would also be highly beneficial for research purposes – for identification of subgroups of children with DLD and accounting for their performance during cognitive and language tasks.

**BOX 1.**

Clinical recommendations ‘To come to tailored assessment and treatment we recommend to assess every individual child with DLD neuropsychologically in addition to their language profiling. Next to linguistic abilities, one should at least zoom in on perception, attention and executive functioning (working memory, inhibition and flexibility). These cognitive domains should be brought to light both behaviourally (e.g., with behavioral rating scales) and cognitively (e.g., with cognitive tests). This is essential not only for clinical purposes (to come to tailored assessment and treatment), but also for experimental studies, when children with DLD are currently treated as a single population while they are likely to represent several clinical groups.’

## Author Contributions

All authors listed have made a substantial, direct and intellectual contribution to the work, and approved it for publication.

## Conflict of Interest Statement

The authors declare that the research was conducted in the absence of any commercial or financial relationships that could be construed as a potential conflict of interest.
